# A Systematic Review of Evidence for the Cost of Therapeutic Resistance in Cancer

**DOI:** 10.64898/2025.12.29.696883

**Published:** 2025-12-31

**Authors:** Bailey Kane, Lauren Mestas, Madds Garza, Tiara Soesilo, Meghan Hufford, Harley Richker, Carlo Maley

**Affiliations:** 1 Arizona Cancer Evolution Center, Arizona State University, Tempe, Arizona, USA 85287-5301; 2 Biodesign Center for Biocomputing, Security and Society, Arizona State University, Tempe, Arizona 85287; 3 School of Life Sciences, Arizona State University, Tempe, Arizona, USA 85281; 4 School of Molecular Sciences, Arizona State University, Tempe, Arizona, USA 85281; 5 Center for Evolution and Medicine, Arizona State University, Tempe, Arizona, 85281

## Abstract

Emergence of therapeutic resistance is a critical clinical challenge in cancer treatment, contributing to treatment failure, disease relapse, and overall poor prognosis. Adaptive therapy (AT), a resistance management strategy, aims to address this issue by selectively applying therapeutic pressure to promote competition between therapy-sensitive and therapy-resistant clones, allowing for long-term control of tumor burden. AT relies upon the assumption that resistance comes at some fitness cost in the absence of therapy. Is that assumption justified? We conducted a systematic review of the literature on experimental tests of the fitness cost of therapeutic resistance. We conducted a search for peer-reviewed papers that fulfilled the following selection criteria: (i) experiments of direct competition, (ii) between therapy-resistant and therapy-sensitive clones, (iii) in a therapy-free environment. We found 47 experiments that matched those criteria. Of those experiments, approximately two-thirds (68%) found a fitness cost to resistance in a competitive environment. Of all pooled features from the studies reviewed, we found that the resistance characteristic was most significantly associated with whether resistant clones exhibited a fitness advantage in competition (p=0.0147). Further, we identify complex ecological interactions that may influence the behavior of the cancer cell population without selection by therapeutic pressure. Predicting which resistance characteristics can be exploited therapeutically with AT and identifying potential methods of modulating the costliness of the resistant phenotype may be critical to future improvements in cancer therapy.

## Lay Summary

When cancer cells evolve to no longer respond to a cancer therapy, do they pay a cost for that invulnerability? We reviewed experiments where cancer cells resistant to a therapy were pitted against cancer cells that were still sensitive to the therapy. Approximately ⅔ of the experiments showed that the sensitive cells could out-compete the resistant cells, implying that the latter were paying some cost for their ability to resist the therapy.

## Overview

### The Clinical Challenge of Resistance in Cancer

Despite major strides in interventional strategies for cancer management, therapeutic resistance represents a major clinical challenge driving treatment failure, disease recurrence, and poor patient outcomes [1]. Standard-of-care approaches are typically administered using a fixed dosing strategy designed to eliminate as many cancer cells as possible within a short window of time. Typically, this dose is determined as the concentration inducing the highest cytotoxicity without inducing intolerable side effects for the patient: the Maximum Tolerated Dose (MTD). MTD treatment often induces appreciable tumor response initially, but can inadvertently enable the selective expansion of therapy-resistant clones that persist through treatment [2]. Thus, the tumor cell population may become refractory to further intervention, leading to malignant recurrence. It is often claimed that 90% of cancer-related deaths are attributed to therapeutic resistance, though the origin of this statistic is unclear. However, there is a broad consensus that the emergence of resistance substantially limits treatment options and remains a barrier to long-term cancer control. Addressing therapeutic resistance, therefore, is critical to the development and refinement of new-generation therapeutics.

### Evolution and Mechanisms of Resistance

Therapeutic resistance is a phenomenon observed across systems [3,4]. Within the scope of this review, therapeutic resistance can be defined as the ability of a tumor to evade treatment due to the failure of neoplastic therapies to eliminate tumor cells.

Primary resistance notwithstanding (Explanatory Box 1), tumors may acquire resistance following a period of therapeutic pressure through two mechanisms: (i) selection for pre-existing resistant clones within a heterogeneous, treatment-naive tumor population, and (ii) the emergence of *de novo* resistant clones from sensitive parental lines [5,6]. Although conceptually distinct, these mechanisms are not mutually exclusive. In addition, resistance characteristics can arise through genetic or non-genetic changes, including reversible phenotypic alterations (phenotypic plasticity), which enable the persistence of drug-tolerant cell states [6,7]. Importantly, both genetic and non-genetic mechanisms can be heritable and therefore relevant to evolution by natural selection [8,9].

Cancer cells employ various resistance strategies that enable them to persist through treatment. At the cellular level, resistance mechanisms include mutational alteration of the target [20,21] and up- or downregulation of the expression of the target [22]. Similarly, pathway bypass can be achieved either by reactivation of the target pathway through downstream alterations or by the activation of compensatory pathways that evade the targeted pathway entirely [23,24].

Drug efflux through ATP-binding cassette (ABC)-family transporters can confer a multidrug-resistant (MDR) phenotype and has been observed across cancer types [25,26]. Metabolic reprogramming, cell death inhibition, and activation of stress responses can mitigate drug-induced cytotoxicity [26]. Additionally, tumor cells may also destroy or inactivate drugs or prevent the conversion of a prodrug to its active form [27]. Further, quiescence is a necessarily costly resistance mechanism to cell-cycle-dependent therapeutic pressures; by halting progression, these cells incur a loss in proliferative capacity, but establish dormancy-like maintenance protocols to withstand treatment and evade the immune system [28].

At the population level, treatment failure and recurrence can also result from inadequate drug delivery due to structural or spatial variation across tumors [29]. Intratumoral heterogeneity (ITH), driven by genomic instability or divergent fitness landscapes, may further drive diversification and lead to the generation of resistant clones as described above [30]. Because cancer cells can employ multiple resistance mechanisms either concurrently or sequentially [31], durable therapeutic control of tumors remains difficult. Thus, treatment strategies that explicitly incorporate eco-evolutionary principles for population management could hold promise in preventing resistance emergence.

### Utility of Evolutionary Strategies for Cancer Management

Natural selection is a fundamental driver of cancer progression [16,32]. The dynamic eco-evolutionary framework of cancer was first conceptualized by Cairns and Nowell in the mid-1970s [33,34]. Resistance management strategies were developed in integrated pest management in the early 1970s [35] and later in infectious disease through antibiotic stewardship initiatives in the 1990s [36]. The guidelines of integrated pest management have since been proposed as universal principles of therapeutic resistance management with potential utility in cancer therapy [4,37].

Adaptive therapy (AT), a resistance management strategy for cancer, was proposed by Gatenby and colleagues in 2009 [38]. Unlike fixed-dose regimens, AT dynamically adjusts treatment intensity in response to measured tumor burden, with the goal of maintaining long-term tumor control rather than maximal tumor eradication [38]. This model assumes two populations: a therapy-responsive, sensitive cell population and a therapy-resistant cell population. It also assumes that there is a cost to therapeutic resistance, such that in the absence of therapy, sensitive cells are expected to out-compete resistant clones. AT leverages therapeutic intervention as negative pressure against sensitive clones, and sensitive clones as a negative pressure against resistant clones, enabling long-term therapeutic management of cancers [39–41,38].

Intermittent or on-off treatment strategies also rely on the presumed cost of resistance. For instance, intermittent androgen deprivation therapy is commonly administered at intermittent doses to prevent or delay androgen insensitivity in prostate cancer [47]. Some metronomic therapies can improve tumor control relative to continuous MTD administration [48]. Some therapy-resistant tumors may regain sensitivity following a drug holiday, consistent with the theory that resistance is selected against in the absence of therapy [49]. While AT shares conceptual foundations with these approaches, it is distinguished from other intermittent treatment strategies by its emphasis on consistent monitoring and real-time dose modulations to dynamically control tumor burden and minimize overall treatment intensity. (Explanatory Box 2).

Although interest in evolution-informed cancer therapy has grown steadily since its conceptual origins in the 1970s, clinical adoption remains limited. This is largely due to the need for stronger clinical evidence and optimized treatment protocols. Early clinical trials have demonstrated the potential of AT in managing reproductive cancers and highlighted challenges in neuroendocrine-type tumors [41,50,51]. Further research is necessary to identify tumor genotypes and phenotypes most amenable to AT and to refine patient-specific treatment parameters in the clinical setting [52].

### Generality of the Assumption of Resistance Cost

Life history theory predicts that investment in defense requires reallocation of resources away from growth and proliferation [53,54]. A model by Hausser et al. (2019) proposes that cancer cells divert resources among five distinct “universal cancer tasks”, with investment constrained by tradeoffs along a continuum of strategies [55].

Further, resistant clones are often present at diagnosis, but typically at frequencies so low as to be rarely detected [13,56,57], suggesting that they do not have a fitness advantage over sensitive clones. If they did, they would tend to have expanded. Cell population sizes are so large, and mutation rates are so high in cancers [32], that they are likely to spontaneously generate resistance mutations, perhaps many times. This perspective suggests that some cases of primary resistance might be cases where a resistant clone had a fitness advantage and expanded to fixation prior to diagnosis, while secondary (acquired) resistance implies a fitness cost of resistance.

Across biological systems, resistance management strategies have proven successful due to the functional compromises that render resistance costly. Fitness costs of resistance have been documented in insects [58], plants [59,60], viruses [61,62], fungi [63], bacteria [64], and parasites [65,66]. In oncology, the assumption of resistance cost is frequently embedded in mathematical models of tumor dynamics [38]. Experimental successes of adaptive therapy, intermittent dosing, and drug-holiday strategies are often attributed, at least in part, to these costs [38,41,43,67]. Given pervasive biological constraints, the existence of resistance-associated tradeoffs is a reasonable and widely invoked assumption.

However, to date, no comprehensive systematic review has evaluated whether therapeutic resistance generally entails a fitness cost. Such costs can be tested through *in vitro* and *in vivo* competition experiments between sensitive and derived resistant cell populations. Currently, there is limited consensus regarding which resistance mechanisms are likely to be costly, the conditions under which these costs emerge, or how environmental context modulates their magnitude. We aim to promote research on predictors of competitiveness of resistant clones and their associated costs that can be exploited by adaptive therapy [43,52].

## Methods

### Search Strategy

This systematic review was conducted in accordance with established guidelines, including PRISMA2020, to define retrieval, screening, and data collection protocols [68,69].

Given the narrow scope of the research question and the variability of keywords used in the literature, a set of thirteen publications identified through a non-systematic search was used as a positive control to inform the construction of itemized search queries. Searches were performed in the databases Scopus, PubMed, and Google Scholar, with database-specific adaptations made to accommodate query language constraints. All search strings are provided in Supplemental Table S1.

Final database searches were conducted on 3 October 2025, yielding 85 records from Scopus, 58 from Google Scholar, and 37 from PubMed. No filters were applied, including restrictions on publication date. To supplement database retrieval, the large language model (LLM)-assisted literature database SciSpace was used to identify additional relevant publications, returning 50 peer-reviewed articles from its associated library. The SciSpace search query is listed in Supplemental Table S1. Citation searching was employed but yielded no additional eligible studies.

### Inclusion Criteria and Screening

After initial retrieval, duplicate records (n=24), abstracts or titles that did not meet eligibility criteria (n=79), publications that were not primary research reports (n=10), and records that could not be retrieved (n=7) were excluded (Supplementary Figure S1). The remaining 119 papers were screened according to the following eligibility criteria: (i) experiments depict direct competition, (ii) between therapy-resistant and therapy-sensitive cancer clones, (iii) in the absence of therapeutic pressure. Among studies addressing therapeutic resistance in cancer (n=56), 23 were excluded for failing to meet all three experimental criteria.

After preliminary full-text eligibility screening, the 44 preliminary papers considered for inclusion were processed through SciSpace with a plain-language prompt (Supplemental Table S1) to search for publications not already captured. No additional papers were retrieved through this step. Final screening measures were conducted by evaluating the experimental design and reviewing supplemental figures. This process led to the exclusion of 22 additional publications. Ultimately, 22 publications that satisfied all inclusion criteria were advanced to the data collection and analysis step.

### Data Collection and Analysis

For each eligible experiment, twenty-two descriptive features were extracted and recorded (Supplemental Table S2). Data items that could not be identified in the main text or within a direct citation were recorded as not available (NA). The complete table of included publications is provided in Supplemental Table S3.

Due to limited sample sizes across categories, statistical analysis of the association between features and the cost of resistance was performed using Fisher’s exact test, implemented via the stats package in R (version 4.5.1).

## Results

### Fitness Differences Between Clones

There were fitness differences between clones in the majority (96%, n=45) of experiments reviewed. Relative success of a clone in competition (“winners” and “losers”) is typically measured by calculated growth rate, death rate, or proportion of the population made up by each clone. However, clonal populations exhibit dynamic behavior that can significantly change winner-loser fates; density-dependent interactions, spatial landscapes, and dynamic phenotypic switching impact competition.

Of 47 separate competition experiments sourced from 22 peer-reviewed publications that met the criteria for inclusion, resistant cells won the competition in 13 (28%) experiments, lost in 32 (68%) experiments, and had neither an advantage nor disadvantage in 2 (4%) experiments. Both the experimental context and the intrinsic properties of the cancer models carry important implications; the former for informing experimental design for future research seeking to address clonal competition in cancer, and the latter for predicting the success of evolutionary-inspired therapies for a particular cancer.

**Figure 3.**
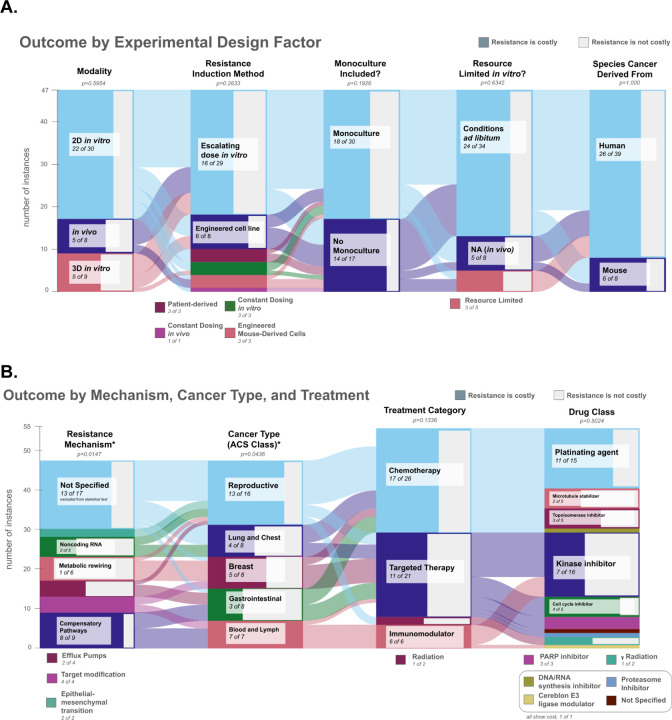
Categorical Variable Association with Experimental Measures of the Cost of Resistance. **A.** Sankey diagram of experimental design variations between experiments by conclusion. Left to right: Experiment modality (p=0.5954), resistance induction method (p=0.2633), monoculture included (p=0.1926), resource limitation (p=0.6342), and species from which the cancer cell line was derived (p=1). None of these features were significantly predictive of a cost of resistance, demonstrating resiliency of clonal competition dynamics across experiments. **B.** Sankey diagram of the mechanisms of resistance, the types of cancer and the types of therapy that generated the resistance. Left to right: resistance mechanism (p=0.0147), Cancer type (American Cancer Society Classifications) (p=0.0436), treatment category (p=0.1336), and class of drug (p=0.5024). For the rightmost two columns, the number of observations increased to 55, as 8 cell lines were resistant to both targeted and chemotherapies and so were counted in both categories. For both A and B, X axis: Number of studies that demonstrated that resistance is costly (colored portion of the bar) or that resistance did not demonstrate a significant cost (gray portion of the bar). Y axis: number of instances in each category. The numbers within each bar provide the number of experiments that demonstrated a cost of resistance out of the total number of experiments in that bar. The Sankey diagram connects studies sharing specified features. Significance values for associations between a given categorical variable and the proportion of experiments that demonstrated a cost of resistance were computed using Fisher’s Exact Test.

In our analysis, two of the eleven categorical features surveyed in each experiment (Supplemental Table S2) were significantly associated with experimental outcome: cancer classification (p=0.0436) and mechanism of resistance (p=0.0147). All other features, including those relating to experimental design rather than features intrinsic to the model, did not demonstrate significance (p>0.05, Figure 3A, B).

### Resistance Characteristics Predict Costliness

Out of all categorical features surveyed, the mechanism of resistance was most significantly associated with experimental outcome in studies that described a specific resistance characteristic. Target modification, EMT and up-regulation of compensatory pathways tended to be associated with a cost of resistance, while resistance via metabolic rewiring was only found to have a fitness cost in 2 out of 6 experiments. Competition results were mixed for resistance due to efflux pumps and non-coding RNAs.

### Target Modification

Two studies in hematopoietic malignancies demonstrated that resistant cells evaded therapy by genetic modification of the target protein. Knockout of *CUL4B*, a gene encoding a subunit of E3 ubiquitin ligase, conferred robust resistance to lenalidomide in an *in vitro* multiple myeloma model from Barrio et al. (2020). Lenalidomide alters the substrate specificity of E3 ubiquitin ligase and induces cell death in lymphocytes [70]. *CUL4B* KO clones had a growth penalty in co-culture against parental cells, suggesting that disruption of the ubiquitination pathway incurs a cost for resistance that is not adaptive in the absence of drug [71]. Further, resistance to the targeted proteasome inhibitor bortezomib in multiple myeloma is conferred by a missense mutation in *PSMB5* near the drug-binding pocket. Alterations in the active site of this proteasome subunit may result in reduced catalytic efficiency, but proliferation was not significantly impacted [72]. Though this mechanism of bortezomib resistance appears to confer little intrinsic fitness penalty, PSMB5 A20T mutants were outcompeted by sensitive clones in drug-free co-culture [73,74]. These data suggest that bortezomib resistance via proteasome binding site modification may incur a fitness penalty that is exploited by sensitive cells.

Target modification may prove costly in breast cancer as well. Resistant cells can escape treatment by the cell-cycle inhibitor NU6102 via mutational modification of cyclin-dependent kinase 2 (CDK2), the target of NU6102. However, resistant cells spend less time in S phase overall and are less fit both in mono- and co-culture compared to parental lines [75].

### Non-coding RNA

Long non-coding RNAs (lncRNA) and microRNAs (miRNA) mediate cell proliferation, motility, and physiology; dysregulation of these systems has been investigated as initiators and drivers of cancer progression [76]. Radiotherapy resistance in PC3 prostate cancer cells was driven by miRNA-95, which drove the activity of sphingolipid phosphatase [77]. These radiotherapy-resistant clones bore a higher proliferation rate than sensitive clones in both monoculture and dominated spheroid co-culture [78]. Success in competition and aggressive phenotype suggest that this mechanism of resistance does not confer a significant fitness cost. Conversely, in models of the prostate cancer line DU145, the radiotherapy-resistant population was driven down by the presence of sensitive cells despite an intrinsic growth advantage. High abundance of the lncRNA UCA-1 was noted in resistant DU145 clones, a mediator of growth and proliferation [79]. Unlike miRNA-95-overexpressing clones, however, the UCA-1-high clones had a fitness disadvantage in competition. This disagreement highlights the differential costs of various resistance mechanisms.

Further, cross-spectrum resistance in non-small cell lung cancer (NSCLC) was conferred by stochastic mutations in *Dicer1*, a key gene involved in miRNA processing. *Dicer1* mutants (“M1”-”M3”), resistant to afatinib, docetaxel, and bortezomib, demonstrated comparable growth rates to parental clones when cultured separately. The resistant “M1” mutant was outcompeted by sensitive clones across a range of seeding densities, but the “M2” and “M3” mutants dominated in co-culture [80].

### Metabolic Rewiring

Lipid metabolism is responsible for the significant competitive edge observed in oxaliplatin-resistant hepatocellular carcinoma (HCC) cells. Resistant cells appear to compensate for the DNA damage induced by oxaliplatin by initiating lipid metabolism and stress pathways mediated by heat shock protein 90 alpha, conferring a significant growth advantage, even in the absence of oxaliplatin [81].

Mitophagy, glycolytic metabolism, and receptor protein tyrosine kinase (RPTK) overexpression appear to confer a growth advantage and lenvatinib resistance in HCC, even in the absence of therapy. Lenvatinib is a multikinase targeted therapy that primarily blocks angiogenesis [82]. Resistant clones dominate both *in vitro* competition assays and in mixed tumors injected orthotopically in mouse models. However, tumors consisting of resistant cells only were smaller by volume than those consisting of sensitive cells only, suggesting a potential hurdle to the altered metabolic program in a physiological environment [83]. *In vitro* cultures in this study were maintained in *ad libitum* conditions; it is possible that the altered metabolic demands may become costly in a physiological environment with limited resources.

Corroborating this, cisplatin-resistant cervical cancer cells exhibited low fitness in vivo due to increased intracellular ROS concentrations resulting from glycolytic metabolism. Tumors composed entirely of resistant cells failed to graft in more than half of subjects, had higher apoptosis rates, and were far smaller by volume than those composed of sensitive cells only [43].

### Efflux Pumps and Epithelial-Mesenchymal Transition (EMT)

In breast cancer, clones resistant to the topoisomerase inhibitor doxorubicin displayed an efflux pump-high, glycolytic phenotype. However, the costliness of maintaining this resistant phenotype varied; it proved either disadvantageous or beneficial in co-culture between studies, despite increased resource demand and variable intrinsic fitness consequences [30,84].

Further, the PARP inhibitor olaparib disrupts DNA repair and is particularly effective in BRCA-mutant cancers [85]; however, treatment evasion is often attained through efflux pump overexpression [86,87]. Olaparib-resistant ovarian cancer lines exhibited epithelial-mesenchymal transition (EMT) markers, as well as efflux pump upregulation. In BRCA1-proficient cell lines, sensitive and resistant clones had similar growth rates when cultured separately; in BRCA1-deficient cell lines, however, the olaparib-resistant clones had an intrinsic fitness penalty [88]. In both cell lines, resistant cells were considerably outcompeted by sensitive cells [25,88]. Taken together, these studies demonstrate that the intrinsic costliness of maintaining an efflux pump-high MDR phenotype in ovarian cancer may confer a fitness disadvantage in competition, particularly when it is associated with an EMT phenotype.

### Compensatory Pathway Activation

A panel of *NRAS* mutations renders myeloproliferative neoplasms resistant to ruxolitinib, a JAK inhibitor. Mutations in *RAS* are the most recognized oncogenic drivers across cancers, and offer an opportunity for cells to recover fitness deficits induced by JAK inhibition by employing non-JAK/STAT-mediated signaling pathways instead [89]. Despite pathway bypass conferring resistance, ruxolitinib-resistant cells were outcompeted in drug-free conditions in both *in vitro* and *in vivo* models. Similarly, mutations in the *Apc* tumor suppressor gene unconstrain *Wnt* signalling, another oncogenic driver across cancers [90,91]. Cells dependent on accelerated *Wnt* signalling are negatively selected in the absence of therapy and lose in competition with sensitive cells *in vivo* [92]. This phenomenon is termed oncogene overdose: cell death or growth arrest triggered by overreliance on an aberrant oncogenic signalling pathway [93]. In these studies probing *RAS* and *Wnt* mutations, oncogene overdose may contribute to decreased fitness in the absence of therapy [94].

Resistant breast cancer cells compensate for the cytotoxicity of ribociclib, a CDK4/6 inhibitor, by increasing estradiol production, which drives growth signaling. Both sensitive and estradiol-overproducing resistant clones have comparable growth rates when cultured separately. However, resistant cells in co-culture promote sensitive cell growth through secreted estradiol production, resulting in a net fitness advantage to the sensitive population and subsequent suppression of resistant cells [95].

Comprehensive review of the fitness consequences of specific resistance characteristics is limited by the small number of studies present in each category as well as the lack of an explicit resistance mechanism described in seven of twenty-two studies. However, general principles appear to underlie competition between therapy-sensitive and therapy-resistant clones reliant upon similar resistance characteristics across cancer types and treatment pressures. This significant association between mechanism of resistance and relative fitness has been demonstrated previously in antimicrobial resistance literature [64], and we are excited to report trends that may suggest the same in cancer.

### Resistance Across Cancer Types

All seven of the competition experiments in hematopoietic cancers found a fitness cost of resistance, and 13 out of 16 experiments in reproductive cancers also found a fitness cost of resistance. Results were more mixed for other types of cancers, with gastrointestinal cancers showing the lowest frequency (3 of 8 experiments) of a fitness cost of resistance (Figure 3).

## Discussion

Fitness of a mutation or phenotype is a complex phenomenon, dependent on the microenvironmental context, the genomic background, and the competitors (as well as the cooperators) in that environment. The experimental tests of the fitness of resistant clones to date are too few to be able to control for the variation introduced by different genomic backgrounds, different tissues of origin, and varying microenvironments, let alone the specific mechanisms of resistance. By grouping studies by the different classes of mechanisms of resistance (in the 30 experiments that described those mechanisms) and by cancer type, we were able to detect some patterns among the 47 competition experiments we found. In addition, the experiments highlighted several important complexities in measuring the cost of therapeutic resistance.

### Intrinsic and Competition-dependent Fitness Consequences

Fitness measures in monoculture often differed from fitness measures in co-culture. Intrinsic, or cell-autonomous, fitness consequences impose a constant burden on the cell, regardless of its microenvironment. On the other hand, competition-dependent, or non-cell-autonomous, fitness consequences are modulated by the microenvironment, including interactions between cells; the fitness gap between cell types is a relevant feature, as are the relative population densities [96,97]. The traditional Lotka-Volterra model, used to represent competition in ecology, includes both intrinsic growth rates and carrying capacities for each population, as well as their competitive effects on each other [98]. Data from monoculture studies can be used to estimate the intrinsic growth rate and carrying capacity for the sensitive and resistant cells.

The combined data from mono- and co-culture can inform the use of ecological frameworks to describe interactions between clones (Explanatory Box 3). Divergence from the predictions of the Lotka-Volterra model (Explanatory Box 3) can reveal complex competition-dependent fitness consequences in co-culture which include facilitation, frequency dependent selection, phenotypic shifts, and cell-cell fusion.

#### I. Facilitation.

The ecological definition of facilitation broadly includes all interactions that result in a fitness benefit for at least one participant [104]. Thus, mutualistic, commensal, and antagonistic interactions all fall under the umbrella of facilitative interactions. Facilitative interactions between clones were observed under both therapeutic pressure and therapy-free conditions.

Antagonism includes predation, farming, parasitism, and exploitation. It describes interactions where one clone benefits at the explicit expense of another. Under drug-free conditions, sensitive cells can derive an additional fitness benefit from the presence of loser resistant lines, mediated by cell-cell contact or paracrine signaling [102,105]. Conversely, resistant clones can derive an additional fitness benefit from competition through upregulated glycolytic metabolism, absent in clones from monocultures [83].

No significant synergistic growth effects (mutualism) in drug-free co-culture were reported in the reviewed studies, but this scenario is theoretically possible, and researchers have begun exploring the consequences of cooperation and altruism in cancer [106].

#### II. Phenotypic shifts in drug response.

Phenotypic shifts modulate the fitness differential between clones and drive the population toward homogeneity in two major ways under drug pressure: by improving resistant cells’ response to therapy (resensitization), or increasing sensitive cells’ tolerance to therapy (desensitization).

Desensitization of sensitive clones by the presence of resistant clones was observed in various models of competition. The ability to transiently adopt resistant phenotypes has significant implications for the success of adaptive therapy [45]. However, the cost assumption by these phenotypic transitions differs mechanistically between models; for instance, microvesicle-mediated horizontal transfer of drug efflux pumps confers a fitness benefit to treatment-naive cells under therapeutic pressure [107]. However, the cost of maintaining the resultant MDR phenotype falls to the recipient due to the significant metabolic burden of efflux pumps. Sensitive cells in this model fall back to baseline drug tolerance levels following cessation of treatment, shedding the high-cost phenotype. Alternatively, tolerance can be induced in the sensitive population by paracrine signalling of growth factors from resistant cells. In this model, sensitive cells benefit from the production of growth factors in the microenvironment without resource expenditure, while the donor population shoulders the cost [95,108]. Craig et al. also propose a phenomenon termed cooperative adaptation to therapy (CAT), wherein resistant clones appear to induce phenotypic transitions that exceed the tolerance observed in monoculture drug pressure through cell-cell contact [80].

Alternatively, effective resensitization of clones was also observed in co-culture. Secreted components from sensitive clones can reduce the competence of resistant clones under therapy [81,109]. Interestingly, one model also demonstrated that sensitive clones may maintain their fitness advantage even under sublethal therapeutic conditions [81]. Taken together, this may suggest that a significant density of therapy-sensitive clones may both exert antagonism against resistant clones and induce phenotypic transitions that favor a sensitive state. This provides further rationale for the maintenance of a population of sensitive clones to combat drug resistance acquisition through dose-modulation adaptive therapy.

#### III. Fusion.

Cell-cell fusion is similar to, but mechanistically distinct from, phenotypic transition in that it is irreversible and results in the effective removal of constituent cells from the population. In an NSCLC model, novel cell-cell nuclear fusion occurred at high rates under drug selection between sensitive and resistant clones, despite a steep competitive edge against chemoresistant clones without therapeutic pressure. The fused cell exhibited greater chemoresistance across a spectrum of agents compared to the parental chemoresistant clone, suggesting not only a novel acclimation to therapy but also an acceleration in the evolution of the mixed population due to genetic heterogeneity [110]. The propagation of drug-resistant clones, however, is stifled by the presence of sensitive clones in absence of therapy; it still stands to reason that even in these rare fusion events, conservative application of therapy could maintain a significant pressure against resistant clones that may drive the resistant population to extinction.

#### IV. Density-Dependent Selection.

Clonal populations can exert and experience fitness consequences according to their relative densities. In one model, alectinib-resistant and sensitive NSCLC parental clones have similar growth rates when cultured separately. In co-culture, however, competition is modulated by drug presence, cancer-associated fibroblasts, and population density. In all conditions but a portion of those cultured with fibroblasts and no drug, parental growth rate is outpaced by that of resistant clones [84]. The major shifts in the growth rate of sensitive and resistant clones observed reinforce that cell-cell interactions can influence the success of phenotypically distinct clones in competition. Further, we identified one case where the winning clone was that which was present in the greater proportion from seeding [39].

### Vulnerabilities and Modulators of Costliness

Fitness is necessarily context-dependent and can vary over space and time [111]. Therefore, it is reasonable to propose that modulation of specific environmental factors might incur increased fitness costs in cells bearing the resistance phenotype, and thereby facilitate therapy.

Efflux pumps pose significant drug delivery challenges across therapy types, but efflux carries a high metabolic demand and therefore may be leveraged for cross-sensitivity [26,112]. Treating MDR clones with a non-cytotoxic efflux pump substrate (called an ersatzdroge) may increase the fitness cost of the resistant cells [84,113,114]. *In vitro* trials have demonstrated the success of ersatzdroges in exhausting MDR clones [84,113]. However, clinical adoption of ersatzdroge-chemotherapy combination treatments failed to demonstrate significant success in eradicating MDR clones [115,116].

The metabolic demands of expensive resistance mechanisms may be exploited in other ways. Resource limitation *in vitro* modulated competition between clones. Although only a small number of studies (n=5, 10%) assessed co-culture in resource-limited conditions, it did not lead to a significantly increased observation of a cost of resistance (Figure 3), though in some cases it did reverse the competitive gap between clones, exposing a cost of resistance [39]. It is difficult to model resource constraints *in vivo*, but there may be methods of “starving” MDR clones via *in situ* metabolic impairment [117]. However, this idea must be approached with caution, as altered glucose availability can paradoxically lead to further dedifferentiation of neoplastic cells and is not universally effective in curbing the proliferation of resistant clones [84,118].

Another concept in evolutionary oncology is evolutionary herding, or the Sucker’s Gambit. This strategy proposes that tumors could be steered into predictable evolutionary states by applying selective pressures that render the population vulnerable to therapy or other exploits [119]. Clinical trials have been proposed to evaluate the efficacy of an initial treatment in enhancing response to a subsequent one, but there have been few tests of this idea [120]. This approach is supported by the possibility that clones at various stages of resistance induction may expose cross-sensitivities that could be exploited by therapy [6,121]. Controlled initiation of oncogene overdose via metronomic therapy has also been demonstrated to prolong tumor control [122].

## Concluding Remarks

Across the 47 competition experiments that we found, a cost of resistance was common (68%), but not universal. The lack of a cost of resistance in the remaining cases does not appear to be explainable by the abundance of resources in the co-culture experiments. Two of the five experiments that tested restricting resources still found no evidence of a cost of resistance, though in all of the remaining experiments, they did find that resistant cells that could win competition in rich media lost competition in resource-restricted media [39]. With a limited sample size (n = 47), it is challenging to reliably determine the effects of various relevant factors, such as the background genotypes in which resistance evolves, the likelihood of a cost of resistance to any particular drug or therapy, let alone a drug class.

In the studies reviewed, we specifically find that employing compensatory pathways may force cells into fitness-compromising states, exposing tradeoffs that may be exploited by AT. We were surprised to find that resistance due to modification of the drug target also tended to impose a fitness cost in all cases reviewed. Conversely, metabolic shifts are frequently observed among resistant clones that maintain a competitive advantage against therapy-responsive clones even in the absence of therapy. Despite the high expense of MDR via efflux pumps, fitness costs were not universal among reviewed studies; however, alterations in the tumor microenvironment, like resource restriction, may increase the fitness penalty of MDR. Our findings corroborate work on tracing acquired resistance development and present interventional opportunities to delay or halt this process [7,12]. In a recent preprint, Gallagher et al. (2025) propose patient-specific “mathematical biomarkers” for the success of AT: estimates of clonal competition based on tumor response to an initial cycle of therapy. In conjunction with these sophisticated modeling techniques and measurable biomarkers as a proxy for tumor burden, the mechanistic data we review here can facilitate the development of AT techniques in the clinical setting [113]. Additionally, the fitness consequences of some mechanisms of resistance, such as amplification of the target or enzymatic drug inactivation, have not been evaluated and should be further investigated.

Therapeutic resistance does not always impose a fitness cost, which presents a challenge for AT; however, a cost of resistance is not the only predictor of success in AT [30,45,46]. Our results, though based on a limited number of experiments, suggest that AT may be more effective in hematopoietic and reproductive cancers, but potentially less effective in gastrointestinal cancers. Significant work toward understanding the cases in which AT is unsuitable is needed.

Here, we present empirical evidence of the fitness cost of resistance in cancer and the patterns that exist in our limited purview. We intend for this review to help oncologists approach the clinical problem of therapeutic resistance, facilitate the development of therapies that avoid or delay the evolution of resistance, and address open questions in the field of evolutionary oncology [52].

## Supplementary Material

Supplement 1

Supplement 2

## Figures and Tables

**Figure 4.**
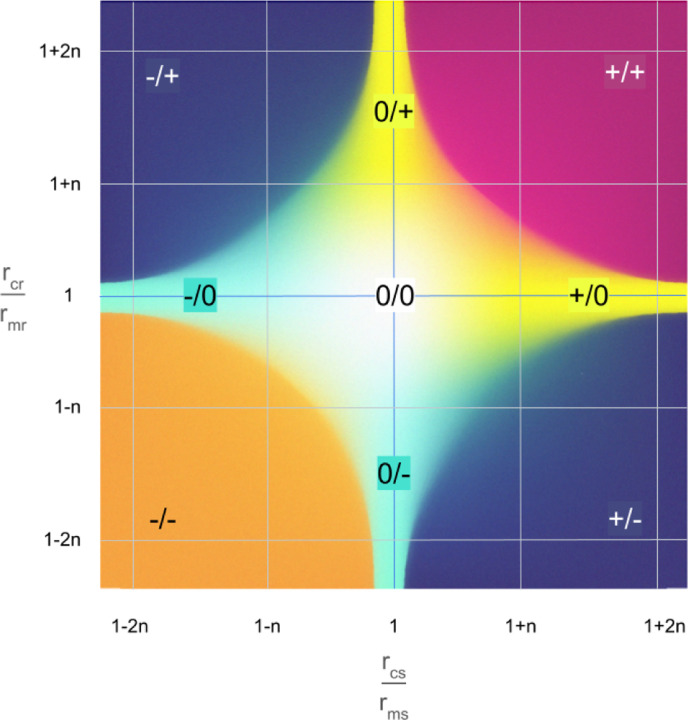
Non-cell-autonomous fitness consequences and ecological interactions. X axis: sensitive population growth rate in co-culture (𝑟_𝑐𝑠_) normalized to rate in monoculture (𝑟_𝑚𝑠_). Y axis: resistant population growth rate in co-culture (𝑟_𝑐𝑟_) normalized to rate in monoculture (𝑟_𝑚𝑟_). For each pair of experiments, there exists a line on this graph where 𝑟_𝑐𝑠_ = 𝑟_𝑐𝑟_, above which resistant clones have a fitness advantage over sensitive clones; below which, vice versa. The quadrants and axes of the figure are labeled with the effects (+, -, or 0) on population growth rate of being in co-culture on the two cell lines. So +/+ means that both cell lines gain a growth benefit of being in co-culture, compared to being in monoculture.

## Data Availability

The data presented in this review are available in the article and in its online supplementary material.

## Explanatory Box 1. Primary and Acquired Resistance

Primary resistance is a flexible term often used to describe both tumors and the individual cells that comprise them, though a tumor may appear to be resistant even if it initially responds to the drug but regrows before the therapeutic effects are measured. For the purpose of this review, we define primary resistance as the inherent tolerance of a cancer to a particular treatment, which presents clinically as nonresponse to treatment. Acquired resistance, on the other hand, is the emergence of resistance over the course of treatment that presents clinically as a gradual inefficacy of therapy following an initial response [10–12]. The confusion arises as such: acquired resistance of a cancer can result from selection on cells bearing preexisting resistance characteristics, which may well be considered primary resistance by some definitions. To avoid this mischaracterization, the terms primary and acquired resistance will refer only to entire tumors, and we will discuss the evolutionary mechanisms by which cancer cells possessing resistant phenotypes are driven to a majority.

Combined with distinct evolutionary histories, the interaction of genetic and non-genetic factors generates various scenarios that explain the persistence of a resistant residual population following treatment. In the traditional view of Darwinian selection, pre-existing resistant clones in a heterogeneous population are capable of withstanding the therapy, producing therapy-tolerant offspring that clonally expand and lead to population-level resistance [13]. In a view of Lamarckian induction, however, a population of cancer cells may contain no pre-existing resistant individuals; instead, the resistant phenotype emerges as a result of gradual phenotypic habituation to therapy [14,15]. In a third scenario of dynamic fluctuation, plastic cells may transiently express a resistant phenotype and persist through treatment (called “persister cells”), but may variably switch phenotypes and shed the putative high-cost resistance program upon cessation of treatment [16,17]. A portion of these cells may enter a fixed resistant state [18]. Each model addresses how a population survives the evolutionary bottleneck imposed by therapy [12], but they are not mutually exclusive; tracing the evolutionary history of cancers reveals that multiple programs may be employed over the course of resistance acquisition [19].

## Explanatory Box 2. Theory of Adaptive Therapy

Adaptive therapy leverages competitive interactions between therapy-sensitive and therapy-resistant clones to keep populations of resistant clones at a controlled, low level. As previously stated, MTD approaches exert negative selection on drug-sensitive cells; in the absence of competitive pressure, resistant clones are free to proliferate and drive clonal expansion. This phenomenon is termed competitive release [42].

Adaptive therapy largely relies on the assumption that maintaining a resistant phenotype confers an intrinsic fitness cost to the cell, and that resistance is adaptive only under therapeutic pressure. In the absence of pressure, resistant cells are outcompeted by fitter sensitive cells. By modulating dosage to tumor burden (estimated by imaging or biomarkers), AT forces tumor population dynamics into predictable, controllable cycles (Figure 2). In recent years, preclinical and clinical data have demonstrated the success of AT in increasing time to progression, improving overall survival, and reducing drug toxicity [39–41,43,44].

Valcz et al. (2025) suggest that AT strategies may be impeded by non-genetic resistance mechanisms [45]. Such mechanisms may enable the rapid expansion of a resistant clonal population at minimal cost. However, non-genetic resistance mechanisms may still be costly and stable enough to be selected upon [8,9,18]. The point stands, however, that dynamic phenotypic switching can quickly deplete the reserve of sensitive cells, which are leveraged to stifle the growth of resistant clonal populations. Conversely, it has been proposed through mathematical approaches that AT need not rely on a cost of resistance to be successful, and that regular cell turnover can increase the time to progression in AT over MTD in tumors approaching carrying capacity [46].

## Explanatory Box 3. Intratumoral Ecological Interactions

Monoculture can demonstrate whether bearing the resistance characteristic confers a disadvantage to net proliferation, while co-culture can demonstrate whether bearing the resistance characteristic bears a competitive disadvantage against sensitive clones. Comparing growth rates in monoculture to those in co-culture demonstrates how cell-cell interactions in the microenvironment modulate fitness beyond simple competition dynamics.

The Lotka-Volterra model of competition was developed independently by Alfred Lotka and Vito Volterra in the early 20th century to describe competition dynamics [99,100]. The equations have utility in modeling intratumoral interactions between distinct populations, each with an independent intrinsic growth rate (*r*_*s*_ and *r*_*r*_) and carrying capacity (*K*_*s*_ and *K*_*r*_). The equations for population dynamics of a sensitive (top) and resistant (bottom) population are given as follows:

$$\frac{{dx}_{s}}{dt}=r_{s}x_{s}(1-(\frac{x_{s}+\alpha_{sr}x_{r}}{K_{s}}))$$

$$\frac{{dx}_{r}}{dt}=r_{r}x_{r}(1-(\frac{x_{r}+\alpha_{rs}x_{s}}{K_{r}}))$$

Where x is the population size, and α is the competition coefficient. α_*rs*_ describes the effect that sensitive cells have on resistant cells; α_*sr*_ describes the effect that resistant cells have on sensitive cells. A positive competition coefficient describes a negative fitness effect on the other clone, while a negative competition coefficient describes a positive fitness effect on the other clone (Table 1)

**Table 1.** Ecological Interactions Described by Competition Coefficient (α)

Ecological Interaction	Competition Coefficient (α)	Definition
Mutualism	α_*sr*_ << 0 and α_*rs*_ << 0	Both populations benefit from the presence of the other.
Commensalism	α_*sr*_ << 0 and α_*rs*_ ≈ 0 or α_*sr*_ ≈ 0 and α_*rs*_ << 0	One population experiences a benefit with no effect on the other.
Neutralism	α_*rs*_ ≈ 0 and α_*sr*_ ≈ 0	Both populations are unaffected by the presence of the other.
Amensalism	α_*sr*_ >> 0 and α_*rs*_ ≈ 0 or α_*sr*_ ≈ 0 and α_*rs*_ >> 0	One population experiences a detriment with no effect on the other.
Competition	α_*sr*_ >> 0 and α_*rs*_ >> 0	Both populations experience a detriment from the presence of the other.
Antagonism	α_*sr*_ << 0 << α_*rs*_ or α_*sr*_ >> 0 >> α_*rs*_	One population experiences a benefit at the detriment of the other.

The various ecological interactions can therefore be inferred from the fitness modulation in co-culture. Take, for instance, a clone X that has a population growth rate of 2 doublings per day. Its derived drug-resistant subclone Y has a population growth rate of 1 doubling per day, demonstrating an intrinsic fitness penalty to maintaining the resistant phenotype. However, in co-culture, growth rates are modulated to 2.5 doublings per day and 1.5 doublings per day, respectively. Though clone Y will likely lose in competition for space and resources due to an inherent fitness disadvantage, its growth rate was enhanced by the presence of clone X, implying mutualistic interactions (Quadrant 1 of Fig. 4). While these data can be derived from cellular automata *in silico*, calibration from biological models improves the accuracy of the model [101].

Accounting for carrying capacity for each individual population is a strength of Lotka-Volterra. Competitive interactions are predicted to become more relevant to clonal dynamics as the total population nears carrying capacity [46]. In one study, resource availability was abundant such that clones in co-culture initially underwent logarithmic growth effectively independent of the other, comparable to growth when cultured separately (phase 1). However, as populations neared carrying capacity, resource scarcity prompted competitive interactions (phase 2) [102].

The Lotka-Volterra model assumes that constituent populations do not undergo significant adaptation and that the environment remains unchanged. It also does not represent density-dependent fitness effects, except for the diminishing proliferation rate as populations near carrying capacity. Despite these limitations, this framework is capable of robustly modeling the dynamics of a clonal population aided by biological data on intrinsic fitness [78,101,103].

## Explanatory box 4. Design Considerations for Competition Experiments

Experimental design varied widely between the competition assays reviewed in this study, though of the particular design features reviewed in Figure 3A, none were significantly associated with experimental outcome (p>0.05, Fisher’s Exact Test). This aside, we noted anecdotal differences between experiments that are supported by prior research. We summarize our findings here for those interested in constructing competition assays for therapy-sensitive and therapy-resistant cancer clones.

### Methods of Generating Therapy-Resistant Cell Lines

While the resistance induction method was not significantly predictive of experiment outcome in our review (p=0.2633), there is prior research investigating how the method by which the resistant cell line is generated influences the resistant characteristics borne by the resulting population. A seminal study by Rath et al. (1984) found that various dosing regimens produce resistant clones with distinct mechanisms of resistance and doubling times that varied nearly sevenfold between clones. Replicates exposed to stepwise increases in drug concentration developed resistance via gene amplification of the dihydrofolate reductase gene and had faster doubling times than those derived from constant exposure to lethal drug concentrations, which lacked gene amplification [123]. Subsequent work has established the influence of the mutational background and expression profile of the parental line, dosing regimen, and drug-free maintenance period on the resistance characteristics, such as surface growth receptor upregulation, ECM profile alterations, or activation of stress pathways, employed by the selected therapy-resistant clones [124].

Some canonical resistance-associated mutations arise frequently within and across cancer types [125]. However, evolution is inherently a stochastic process, with the potential for unique resistance characteristics to emerge under identical selective pressures in separate replicates or between patients [88,126,127]. We therefore emphasize the limitations of engineered cell lines or cell lines derived from engineered mice to generate therapy-resistant clones. These engineered models provide insight into specific mutational mechanisms of resistance, but may not recapitulate the non-genetic resistant physiology that emerges from drug-selective methodologies [124].

### Models

In our review, experiment modality did not bear a significant influence on the likelihood that resistance had a fitness cost (p=0.5954). Models are intentionally designed to capture various levels of physiological complexity and therefore serve different use cases. However, clonal population dynamics may shift between models; in one case, a fitness advantage observed *in vitro* for the sensitive population was absent in the corresponding *in vivo* trial [128]. *In vitro* cultures of cancer cells only do not recapitulate the full spectrum of microenvironmental interactions *in vivo*. Some studies present *in vitro* experiments designed with this limitation in mind, and demonstrate that interactions with stromal cells and competition for limited space, oxygen, and nutrients influence the competitive ability of clones [39,78,84,96].

Further, Bacevic and colleagues demonstrated the efficacy of AT in a three-dimensional spheroid model, whereas AT did not prove to be successful in controlling population dynamics in two-dimensional culture [75]. Culture spheroid and *in vivo* tumor models illustrate three-dimensional spatial and colocalization dynamics, which can modulate hypoxia, extracellular architecture, and nutrient availability [129]. Paczkowski et al. found that in co-cultured prostate cancer spheroids, highly proliferative resistant clones constituted much of the periphery, while sensitive parental cells were constrained to the hypoxic spheroid core [78]. Similarly, Thege et al. found that drug flux to the sensitive spheroid core was reduced by surface localization of resistant clones [109]. Spatial dynamics are critical to assessing fitness landscapes relevant to clonal competition.

### Admixture Ratio

The ratio of sensitive to resistant clones utilized in the described experiments can be found in Supplementary Table S3. Most studies elected to use an admixture with sensitive cells in the majority, though the sensitive:resistant cell ratio varied from 99:1 (99% sensitive to 1% resistant) to 1:8 (11% sensitive to 89% resistant) across all studies reviewed. Experimental conclusion was concordant across the cell seeding ratios described in each row of Supplementary Table S3. Only one experiment from Hockings et al. (2025) demonstrated significant density-dependent fitness effects, where the winner clone was the one which was present in a higher proportion at seeding [39].

Experimental design may constrain the conclusions that can be drawn from the results. The variation within co-culture experiments alone underscores the importance of consistency in design when interpreting results within and between studies.
