# Supplementary material for: A Systematic Review of Evidence for the Cost of Therapeutic Resistance in Cancer
Source: bioRxiv. 2025 Dec 31:2025.12.29.696883. Preprint. [Version 1] doi: 10.64898/2025.12.29.696883 (PMC12773004; doi:10.64898/2025.12.29.696883)
Supplement: Supplement 1 [file media-1.pdf]

Supplemental Table S1

Search Strings and Dates Conducted

Database	Date Searched	Query	No. Results
SciSpace	9 September 2025	“What is the fitness cost of drug resistance mutations in cancer cells and how do resistant clones compete with sensitive cells?”	50
Google Scholar	3 October 2025	cancer AND ("therapeutic resistance" OR "drug resistance") AND ("co-culture" OR "coculture" OR "mixed culture" OR "competition assay" OR "clonal competition") AND "fitness" AND (trade*off OR *advantage OR select*) AND ("in vivo" or "in vitro") AND (evolution OR darwin OR selection) AND compete AND select)) -thesis -dissertation -fungal -antibiotic	58
Scopus	3 October 2025	(TITLE-ABS-KEY (("co-culture" OR "coculture" OR "mixed culture" OR "competition assay" OR "clonal mixture" OR "heterogeneous culture") AND ("sensitive" OR "drug-sensitive" OR "therapy-sensitive" OR "chemosensitive") AND ("resistant" OR "drug-resistant" OR "therapy-resistant" OR "chemoresistant") AND ("clonal competition" OR "clonal fitness" OR "cell competition" OR "competitive interactions" OR "cooperative interactions" OR "tumor evolution" OR "adaptive therapy" OR "evolutionary dynamics") AND (cancer OR tumor OR tumour OR neoplasm OR carcinoma)) AND NOT (fungal OR yeast OR bacteria OR antibiotic)) OR (TITLE-ABS-KEY (("drug-resistant" OR "therapy-resistant" OR "chemoresistant") AND ("drug-sensitive" OR "therapy-sensitive" OR "chemosensitive") AND ("cancer" OR "tumor" OR "tumour" OR "neoplasm" OR "carcinoma") AND ("fitness" OR "growth rate" OR "competitive fitness" OR "cell competition" OR "proliferation rate" OR "competitive assay" OR "competition assay" OR "clonal competition" OR "evolutionary dynamics" OR "growth advantage" OR "fitness cost") AND ("cell line" OR "co-culture" OR "coculture" OR "in vitro" OR "culture experiment")) AND NOT (fungal OR yeast OR bacteria OR antibiotic)) OR (TITLE-ABS-KEY (competitive co-culture cancer resistant)) OR (ALL (cancer AND ("therapeutic resistance" OR "drug resistance") AND ("co-culture" OR coculture OR "mixed culture" OR "competition assay" OR "clonal competition") AND fitness AND ("in vivo" OR "in vitro") AND (evolution OR darwin OR selection)) AND NOT ALL (fungal) AND NOT ALL (antibiotic) AND NOT ALL (bacterial)) AND (EXCLUDE (DOCTYPE , "re"))	85
PubMed	3 October 2025	(("clonal competition"[tiab] OR "clonal fitness"[tiab] OR "fitness cost"[tiab] OR "competitive *advantage"[tiab] OR "growth cost"[tiab]) AND ("resistant clones"[tiab] OR "resistance mutations"[tiab] OR "drug resistance"[tiab] OR "therapeutic resistance"[tiab]) AND ("cancer"[tiab] OR "neoplasms"[mesh]))	37
SciSpace	15 October 2025	“Below is a list of 33 papers that have experiments where therapy-sensitive and therapy-resistant cancer cell lines are directly co-cultured or otherwise mixed in a drug-free environment to evaluate fitness consequences of therapeutic resistance. Find related papers that meet this criteria without repeating any on the list. In-vivo or in-vitro models are acceptable.”	0

Supplemental Figure S1

PRISMA2020 Chart (A) and Sankey Diagram (B)

A.

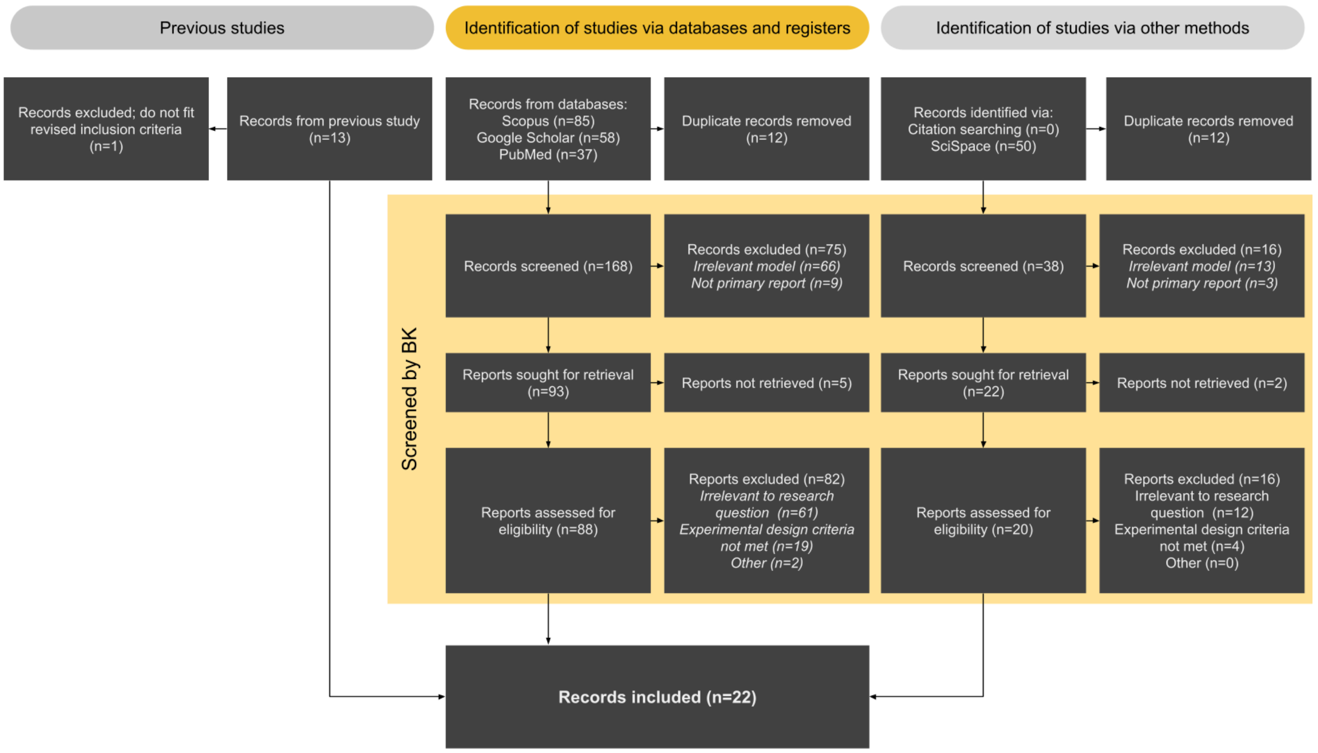

B.

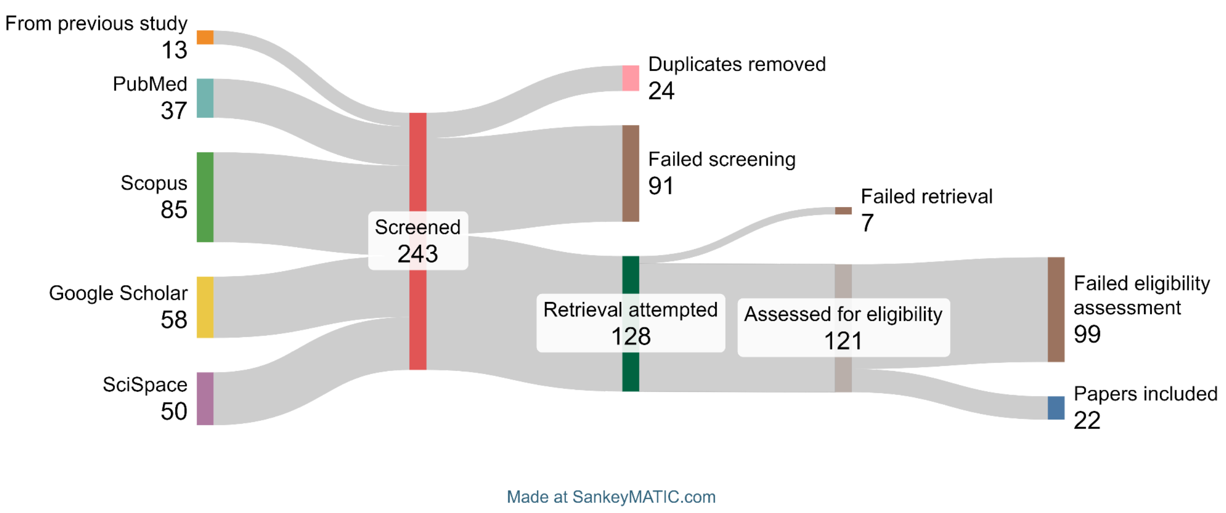

Supplemental Table S2

Data Collection Table Columns

#	Column Name	Description	Data Type	Options
1	Title	Title and citation of the publication	Nominal	*Unconstrained*
2	Experiment number (Exp. #)	If a publication contains multiple experiments; the experiment number	Numerical	1-6
3	Conclusion	The binary winner or loser status of resistant clones: did the resistant line win in co-culture?	Nominal	Resistance does not confer a significant fitness difference<br>Resistance is beneficial<br>Resistance is costly
4	Modality	Describes if the experiment was conducted *in vivo* or *in vitro*	Nominal	*in vitro*<br>*in vivo*
5	Experimental model type	Describes the experimental model used	Nominal	2D co-culture<br>3D co-culture spheroids<br>Orthotopic mouse model
6	Summary	A summary of the experiment	Nominal	*Unconstrained*
7	Measure of fitness	What metric was used to evaluate fitness?	Nominal	Population Growth Rate<br>Cellular proliferation rate<br>Cellular death rate
8	Fitness data collected by	What experimental techniques were used to measure fitness?	Nominal	*Unconstrained*
9	Resource limited?	Was the experiment conducted in resource-limited conditions? (*in vitro* experiments only)	Logical	TRUE<br>FALSE<br>NA
10	Cancer model	Describes the cancer cell line used and what species it is derived from	Nominal	*Unconstrained*
11	Cancer type ACS Class	Class of cancer by American Cancer Society classifications	Nominal	Blood and Lymph<br>Breast<br>Gastrointestinal<br>Lung and Chest<br>Reproductive
12	Sensitive line	Name of the sensitive cell line used	Nominal	NA
13	Resistant line	Name of the resistant cell line used	Nominal	NA
14	Admixture Ratio (Sens:Res)	The simplified ratio(s) of sensitive to resistant cells used in co-culture, if provided	Numerical	NA
15	Resistance Mechanism category	The broad mechanism of resistance category, if provided	Nominal	Compensatory Pathways<br>Efflux Pumps<br>EMT<br>Metabolic Rewiring<br>Target Modulation<br>miRNA Dysregulation
16	Specific resistance characteristic	The specific mechanism of resistance, if identified	Nominal	NA

#	Column Name	Description	Data Type	Options
17	Resistance induction method	The method by which resistant cell lines were generated for use in the study	Nominal	Engineered mouse model-derived<br>*in vitro* constant dosing<br>*in vitro* incremental dosing<br>*in vivo* constant dosing<br>Gene editing<br>Patient-derived
18	Treatment category	The class of treatment to which the resistant line is primarily tolerant; usually the class of drug to which resistance was induced, if applicable	Nominal	Chemotherapy<br>Immunomodulatory Agent<br>Multiple<br>Radiation<br>Targeted Therapy
19	Class of drug	The mechanism of action of the drug	Nominal	Cereblon E3 ligase modulator<br>Proteasome inhibitor<br>Platinating agent<br>Kinase inhibitor<br>Gamma radiation<br>Microtubule stabilizer<br>Topoisomerase inhibitor<br>Cell cycle inhibitor<br>Nucleic acid synthesis Inhibitor<br>PARP Inhibitor
20	Treatment name (trx name)	The specific name of the drug used, if applicable	Nominal	*Unconstrained*
21	Includes monoculture?	Does the study include a complementary experiment with the same parameters where cell lines are cultured separately?	Logical	TRUE<br>FALSE<br>NA
22	Monoculture conclusion	The summary of results from the monoculture experiment	Nominal	*Unconstrained*

Supplemental Table S3

Complete table of included experiments

Title	Exp. #	Conclusion	Modality	Experimental model type	Summary	Measure of fitness	Fitness data collected by	Resource limited?	Cancer model	Cancer Type (ACS Class)	Sensitive line	Resistant line	Admixture ratio (Sens:Res)	Resistance mechanism category	Specific resistance characteristic	Resistance induction method	Treatment category	Class of drug	Trx name	Includes monoculture?	Monoculture Conclusion	Monoculture Summary
IKZF1/3 and CRL4CRBN E3 ubiquitin ligase mutations and resistance to immunomodulatory drugs in multiple myeloma (Barrio et al., 2020)	1	Resistance is costly	*in vitro*	2D co-culture	CUL4B KO conferred lenalidomide resistance to multiple myeloma cells, but a growth penalty in co-culture against parental cells	Population growth rate	Absolute number of cells by flow cytometry	FALSE	Human multiple myeloma	Blood and Lymph	L363 CUL4B WT	L363 CUL4B KO	1:1	Target modification	CUL4B KO	Engineered cell line	Immunomodulatory agent	Cereblon E3 ligase modulator	lenalidomide	FALSE	NA	NA
Clonal competition assays identify fitness signatures in cancer progression and resistance in multiple myeloma (Haertle et al., 2024)	1	Resistance is costly	*in vitro*	2D co-culture	PSMB5 A20T conferred bortezomib resistance to multiple myeloma cells, but a growth penalty in co-culture against parental cells	Population growth rate	Absolute number of cells by flow cytometry	FALSE	Human multiple myeloma	Blood and Lymph	L363 PSMB5 WT	L363 PSMB5 A20T	11:9	Target modification	PSMB5 A20T	Engineered cell line	Targeted therapy	Proteasome inhibitor	bortezomib	FALSE	NA	NA
Adaptive therapy exploits fitness deficits in chemotherapy-resistant ovarian cancer to achieve long-term tumor control (Hockings et al., 2025)	1	Resistance confers benefit	*in vitro*	2D co-culture	Co-culture in high-resource conditions allowed for outcompetition of sensitive clones by cisplatin-resistant clones. Resistance induced **in-vitro.**	Population growth rate	Absolute number of cells by flow cytometry	FALSE	Human high-grade serious ovarian cancer	Reproductive	OVCAR4	Ov4Cis	19:1, 17:3, 3:1, 1:1, 1:3, 3:17, 1:19	NA	NA	Escalating dose In-vitro	Chemotherapy	Platinating agent	cisplatin	TRUE	Resistance confers no significant intrinsic fitness difference	Both cell lines displayed exponential growth in ad libitum conditions
Adaptive therapy exploits fitness deficits in chemotherapy-resistant ovarian cancer to achieve long-term tumor control (Hockings et al., 2025)	2	Resistance is costly	*in vitro*	2D co-culture	In resource-limited conditions, co-culture resulted in outcompetition of cisplatin-resistant clones by sensitive clones. Greater rates of apoptosis and cell cycle arrest in resistant pop in coculture compared to monoculture. Resistance induced **in-vitro.**	Population growth rate	Absolute number of cells by flow cytometry	TRUE	Human high-grade serious ovarian cancer	Reproductive	OVCAR4	Ov4Cis	17:3, 1:1, 3:17	NA	NA	Escalating dose In-vitro	Chemotherapy	Platinating agent	cisplatin	TRUE	Resistance confers no significant intrinsic fitness difference	Both cell lines displayed reduced, but similar to one another, growth rates in limited-resource conditions
Adaptive therapy exploits fitness deficits in chemotherapy-resistant ovarian cancer to achieve long-term tumor control (Hockings et al., 2025)	3	Resistance is costly	*in vitro*	2D co-culture	In resource-limited conditions, co-culture resulted in outcompetition of carboplatin-resistant clones by sensitive clones. Resistance induced **in-vitro.**	Population growth rate	Absolute number of cells by flow cytometry	TRUE	Human high-grade serious ovarian cancer	Reproductive	OVCAR4	Ov4Carbo	17:3, 1:1, 3:17	NA	NA	Escalating dose In-vitro	Chemotherapy	Platinating agent	carboplatin	TRUE	Resistance confers no significant intrinsic fitness difference	"mean subcutaneous tumor growth was comparable in OVCAR4 and Ov4Carbo cells although growth was variable between individual tumors"
Adaptive therapy exploits fitness deficits in chemotherapy-resistant ovarian cancer to achieve long-term tumor control (Hockings et al., 2025)	4	Resistance is costly	*in vitro*	2D co-culture	In resource-limited conditions, co-culture resulted in outcompetition of carboplatin-resistant clones by sensitive clones. Resistance induced **in-vivo.**	Population growth rate	Absolute number of cells by flow cytometry	TRUE	Human high-grade serious ovarian cancer	Reproductive	OVCAR4	IVR01	17:3, 1:1, 3:17	NA	NA	Constant dosing In-vivo	Chemotherapy	Platinating agent	carboplatin	FALSE	NA	NA
Adaptive therapy exploits fitness deficits in chemotherapy-resistant ovarian cancer to achieve long-term tumor control (Hockings et al., 2025)	5	Resistance does not confer a significant fitness difference	*in vitro*	2D co-culture	In resource-limited conditions, co-culture resulted in outcompetition of whichever clone was present in the lesser proportion. Resistance induced **in-vitro.**	Population growth rate	Absolute number of cells by flow cytometry	TRUE	Human high-grade serious ovarian cancer	Reproductive	Cov318	Cov-Cis	17:3, 1:1, 3:17	NA	NA	Escalating dose In-vitro	Chemotherapy	Platinating agent	cisplatin	FALSE	NA	NA
Adaptive therapy exploits fitness deficits in chemotherapy-resistant ovarian cancer to achieve long-term tumor control (Hockings et al., 2025)	6	Resistance is costly	*in vivo*	Orthotopic mouse model	When mixed cultures of cisplatin-sensitive and -resistant clones were injected into mice, the proportion of resistant cells was decreased at 12 weeks. Regions of tumor derived from resistant cells had high Cas3 cleavage.	Population growth rate, Cellular proliferation rate, Cellular death rate	Amount of tag DNA by qPCR and approximate clonal contribution by IHC and pixel quantification	NA	Human high-grade serious ovarian cancer	Reproductive	OVCAR4-GFP	Ov4Cis-RFP	4:1, 1:1, 1:9	NA	NA	Escalating dose In-vitro	Chemotherapy	Platinating agent	cisplatin	FALSE	NA	NA
Evolution of Relapse-Proficient Subclones Constrained by Collateral Sensitivity to Oncogene Overdose in Wnt-Driven Mammary Cancer (Keller and Gunther, 2019)	1	Resistance is costly	*in vivo*	Orthotopic mouse model	Oncogene-addicted resistant lines cells were outcompeted by sensitive cells when targeted therapy was withheld in a Wnt-driven model of mouse mammary carcinoma	Population growth rate	Approximate clonal contribution by MRS-informed tumor segmentation	NA	Mouse mammary carcinoma	Breast	P (iWnt/ApcMin parental mammary tumor)	R2 (iWnt/ApcMin mammary tumor relapse)	NA	Compensatory pathways	APCmmcr 1568*/1522*, APCmmcr 1568+T, APCmmcr 1522 A>T	Engineered mouse model-derived	Targeted therapy	NA	NA	TRUE	Resistance confers no significant intrinsic fitness difference	Homotypic injection of R2+R1 lines results in no significant selection on a line.
JAK2 inhibition mediates clonal selection of RAS pathway mutations in myeloproliferative neoplasms (Maslah et al., 2025)	1	Resistance is costly	*in vitro*	2D co-culture	Nras-mutant, ruxolitinib-resistant mouse bone marrow cells were outcompeted by sensitive cells in drug-free vehicle (DMSO) conditions	Population growth rate	Absolute number of cells by flow cytometry	FALSE	Mouse myeloproliferative neoplasm	Blood and Lymph	NRAS WT Lin- bone marrow cells	NRAS G12D Lin- bone marrow cells	1:1, 4:1	Compensatory pathways	NRAS G12D	Engineered mouse model-derived	Immunomodulatory agent	Kinase inhibitor	ruxolitinib	FALSE	NA	NA
JAK2 inhibition mediates clonal selection of RAS pathway mutations in myeloproliferative neoplasms (Maslah et al., 2025)	2	Resistance is costly	*in vivo*	Orthotopic mouse model	Nras-mutant, ruxolitinib-resistant mouse bone marrow cells were outcompeted by empty casette, WT sensitive cells in drug-free vehicle (DMSO) conditions	Population growth rate	Absolute number of cells by flow cytometry	NA	Mouse myeloproliferative neoplasm	Blood and Lymph	NRAS WT Lin- bone marrow cells	NRAS G12D Lin- bone marrow cells	9:1	Compensatory pathways	NRAS G12D	Engineered mouse model-derived	Immunomodulatory agent	Kinase inhibitor	ruxolitinib	FALSE	NA	NA
JAK2 inhibition mediates clonal selection of RAS pathway mutations in myeloproliferative neoplasms (Maslah et al., 2025)	3	Resistance is costly	*in vitro*	2D co-culture	Nras-mutant, ruxolitinib-resistant human bone marrow cells were outcompeted by sensitive cells in drug-free vehicle (DMSO) conditions	Population growth rate	Absolute number of cells by flow cytometry	FALSE	Human myeloproliferative neoplasm	Blood and Lymph	HEL NRAS WT	HEL NRAS Q61K	4:1	Compensatory pathways	NRAS Q61K	Engineered cell line	Immunomodulatory agent	Kinase inhibitor	ruxolitinib	FALSE	NA	NA
JAK2 inhibition mediates clonal selection of RAS pathway mutations in myeloproliferative neoplasms (Maslah et al., 2025)	4	Resistance is costly	*in vitro*	2D co-culture	Nras-mutant, ruxolitinib-resistant human bone marrow cells were outcompeted by sensitive cells in drug-free vehicle (DMSO) conditions	Population growth rate	Absolute number of cells by flow cytometry	FALSE	Human myeloproliferative neoplasm	Blood and Lymph	UKE-1 NRAS WT	UKE-1 NRAS Q61K	4:1	Compensatory pathways	NRAS Q61K	Engineered cell line	Immunomodulatory agent	Kinase inhibitor	ruxolitinib	FALSE	NA	NA
JAK2 inhibition mediates clonal selection of RAS pathway mutations in myeloproliferative neoplasms (Maslah et al., 2025)	5	Resistance is costly	*in vitro*	2D co-culture	Nras-mutant, ruxolitinib-resistant human bone marrow cells were outcompeted by sensitive cells in drug-free vehicle (DMSO) conditions	Population growth rate	Absolute number of cells by flow cytometry	FALSE	Humanized mouse pro-b cell	Blood and Lymph	Ba/F3 NRAS WT	Ba/F3 NRAS Q61K	4:1	Compensatory pathways	NRAS Q61K	Engineered cell line	Immunomodulatory agent	Kinase inhibitor	ruxolitinib	FALSE	NA	NA

Title	Exp. #	Conclusion	Modality	Experimental model type	Summary	Measure of fitness	Fitness data collected by	Resource limited?	Cancer model	Cancer Type (ACS Class)	Sensitive line	Resistant line	Admixture ratio (Sens:Res)	Resistance mechanism category	Specific resistance characteristic	Resistance induction method	Treatment category	Class of drug	Trx name	Includes monoculture?	Monoculture Conclusion	Monoculture Summary
BNIP3-mediated mitophagy boosts the competitive growth of Lenvatinib-resistant cells via energy metabolism reprogramming in HCC (Wang et al., 2024)	1	Resistance confers benefit	*in vitro*	2D co-culture	Overexpression of RPTKs and glycolytic enhancement conferred both lenvatinib resistance and a growth advantage in vitro.	Population growth rate	Absolute number of cells by flow cytometry and high-content imaging	FALSE	Human hepatocellular carcinoma	Gastrointestinal	CCHuh7m	CCHuh7R	1:1	Metabolic rewiring	RPTK hi	Escalating dose In-vitro	Targeted therapy	Kinase inhibitor	lenvatinib	TRUE	Resistance is intrinsically beneficial	Sensitive Huh7 lines had decreased growth in competitive co-culture compared to noncompetitive mono- or co-culture. Further, sensitive cells from competitive co-culture assays retained a growth disadvantage even in monoculture, though sensitive cells from noncompetitive cultures retained baseline proliferation rates. Monoculture of resistant line demonstrated increased proliferation rate compared to monoculture baseline proliferation for sensitive lines.
BNIP3-mediated mitophagy boosts the competitive growth of Lenvatinib-resistant cells via energy metabolism reprogramming in HCC (Wang et al., 2024)	2	Resistance confers benefit	*in vivo*	Orthotopic mouse model	RPTK-overexpressing lines with glycolytic enhancement conferred lenvatinib resistance and a competitive growth advantage in vivo	Population growth rate	Number of cells by tumor volume, approximate clonal contribution by fluorescence imaging and H&E stain, and absolute number of cells by flow cytometry and high-content imaging	NA	Human hepatocellular carcinoma	Gastrointestinal	CCHuh7m	CCHuh7R	1:1	Metabolic rewiring	RPTK hi	Escalating dose In-vitro	Targeted therapy	Kinase inhibitor	lenvatinib	TRUE	Resistance is intrinsically costly	Huh7R cells alone yielded smaller tumors by volume.
BNIP3-mediated mitophagy boosts the competitive growth of Lenvatinib-resistant cells via energy metabolism reprogramming in HCC (Wang et al., 2024)	3	Resistance confers benefit	*in vitro*	2D co-culture	Overexpression of RPTKs and glycolytic enhancement conferred both lenvatinib resistance and a growth advantage in vitro	Population growth rate	Absolute number of cells by flow cytometry and high-content imaging	FALSE	Human hepatocellular carcinoma	Gastrointestinal	CCPLC-PRF-5m	CCPLC-PRF-5R	1:1	Metabolic rewiring	RPTK hi	Escalating dose In-vitro	Targeted therapy	Kinase inhibitor	lenvatinib	TRUE	Resistance confers no significant intrinsic fitness difference	mCherry-tagged sensitive HCC cell lines had decreased growth in competitive co-culture compared to noncompetitive monoculture
BNIP3-mediated mitophagy boosts the competitive growth of Lenvatinib-resistant cells via energy metabolism reprogramming in HCC (Wang et al., 2024)	4	Resistance confers benefit	*in vivo*	Orthotopic mouse model	RPTK-overexpressing lines with glycolytic enhancement conferred lenvatinib resistance and a competitive growth advantage in vivo	Population growth rate	Number of cells by tumor volume, approximate clonal contribution by fluorescence imaging and H&E stain, and absolute number of cells by flow cytometry and high-content imaging	NA	Human hepatocellular carcinoma	Gastrointestinal	CCPLC-PRF-5m	CCPLC-PRF-5R	1:1	Metabolic rewiring	RPTK hi	Escalating dose In-vitro	Targeted therapy	Kinase inhibitor	lenvatinib	TRUE	Resistance is intrinsically costly	PLC-PRF-5R cells alone yielded smaller tumors by volume.
Cell facilitation promotes growth and survival under drug pressure in breast cancer (Emond et al., 2023)	1	Resistance is costly	*in vitro*	3D co-culture spheroids	Growth rate of ribociclib-resistant CAMA-1 clones is reduced in co-culture while sensitive clones see a growth advantage due to increased estradiol production by resistant clones.	Population growth rate	Approximate clonal contribution by fluorescence imaging	FALSE	Human ER+ breast cancer	Breast	CAMA-1 ribociclib-sensitive	CAMA-1 ribociclib-resistant	1:1	Compensatory pathways	Estradiol production hi	Escalating dose In-vitro	Targeted therapy	Cell cycle inhibitor	ribociclib	TRUE	Resistance confers no significant intrinsic fitness difference	Sensitive cells in monoculture had a slower growth rate than when co-cultured, suggesting a winner/loser dynamic. Resistant cells in monoculture have a similar growth rate to sensitive cells.
Cell facilitation promotes growth and survival under drug pressure in breast cancer (Emond et al., 2023)	2	Resistance is costly	*in vitro*	3D co-culture spheroids	Growth rate of ribociclib-resistant LY2 clones is reduced in co-culture while sensitive clones see a growth advantage.	Population growth rate	Approximate clonal contribution by fluorescence imaging	FALSE	Human ER+ breast cancer	Breast	LY2 ribociclib-sensitive	LY2 ribociclib-resistant	4:1	Compensatory pathways	Estradiol production hi	Escalating dose In-vitro	Targeted therapy	Cell cycle inhibitor	ribociclib	TRUE	Resistance confers no significant intrinsic fitness difference	Both cell lines had similar growth rates in absence of therapy.
Cell facilitation promotes growth and survival under drug pressure in breast cancer (Emond et al., 2023)	3	Resistance confers benefit	*in vitro*	3D co-culture spheroids	Growth rate of ribociclib-resistant MCF7 clones is increased in co-culture while sensitive clones see a growth disadvantage.	Population growth rate	Approximate clonal contribution by fluorescence imaging	FALSE	Human ER+ breast cancer	Breast	MCF7 ribociclib-sensitive	MCF7 ribociclib-resistant	9:1	Compensatory pathways	Estradiol production hi	Escalating dose In-vitro	Targeted therapy	Cell cycle inhibitor	ribociclib	TRUE	Resistance is intrinsically beneficial	Resistant cell lines had highr growth rates than sensitive lines when cultured separately.
Reciprocal interactions between tumour cell populations enhance growth and reduce radiation sensitivity in prostate cancer (Paczkowski et al., 2021)	1	Resistance confers benefit	*in vitro*	3D co-culture spheroids	Growth rate of radiation-resistant PC3 cells is greater than that of rad-sensitive clones	Population growth rate, Cellular proliferation rate, Cellular death rate	Absolute number of cells by flow cytometry, proliferation rate by EdU assay, death rate by flow cytometry	FALSE	Human prostate cancer	Reproductive	PC3 Parental	PC3 RR	1:1	Noncoding RNA	miR-95 upregulation targeting SGPP1	Escalating dose In-vitro	Radiation	Gamma radiation	NA	TRUE	Resistance is intrinsically beneficial	Monoculture sensitive spheroids were smaller than monoculture resistant spheroids, and sensitive cells have lower growth rates in

Title	Exp. #	Conclusion	Modality	Experimental model type	Summary	Measure of fitness	Fitness data collected by	Resource limited?	Cancer model	Cancer Type (ACS Class)	Sensitive line	Resistant line	Admixture ratio (Sens:Res)	Resistance mechanism category	Specific resistance characteristic	Resistance induction method	Treatment category	Class of drug	Trx name	Includes monoculture?	Monoculture Conclusion	Monoculture Summary
																						monoculture
Reciprocal interactions between tumour cell populations enhance growth and reduce radiation sensitivity in prostate cancer (Paczkowski et al., 2021)	2	Resistance is costly	*in vitro*	3D co-culture spheroids	Growth rate of radiation-resistant DU145 cells is reduced compared to sensitive lines	Population growth rate	Absolute number of cells by flow cytometry	FALSE	Human prostate cancer	Reproductive	DU145 Parental	DU145 RR	1:1	Noncoding RNA	lncRNA UCA1 upregulation	Escalating dose In-vitro	Radiation	Gamma radiation	NA	TRUE	Resistance is intrinsically beneficial	Monoculture sensitive spheroids were smaller than monoculture resistant spheroids, and sensitive cells have lower growth rates in monoculture
Fibroblasts and alectinib switch the evolutionary games played by non-small cell lung cancer (Kaznatcheev et al., 2019)	1	Resistance confers benefit	*in vitro*	2D co-culture	In drug-free conditions, resistant clones have an increased growth rate compared to parental lines, despite the two having similar growth rates when cultured separately.	Population growth rate	Approximate clonal contribution by fluorescence imaging	FALSE	Human non-small cell lung cancer	Lung and Chest	H3122 Parental	H3122 Resistant	9:1, 4:1, 3:2, 2:3, 1:4, 1:9	NA	NA	Escalating dose In-vitro	Targeted therapy	Kinase inhibitor	alectinib	TRUE	Resistance confers no significant intrinsic fitness difference	Resistant and parental lines have similar growth rates in monoculture.
Cooperative adaptation to therapy (CAT) confers resistance in heterogeneous non-small cell lung cancer (Craig et. al, 2019)	1	Resistance is costly	*in vitro*	3D co-culture spheroids	In co-culture, resistant Dicer1 mutant cells have a slower growth rate than sensitie parental cells	Population growth rate	Approximate clonal contribution by fluorescence imaging and absolute number of cells by flow cytometry	FALSE	Mouse non-small cell lung cancer	Lung and Chest	Dicer1 WT	Dicer1 M1 mutant	9:1, 1:1, 1:9	Noncoding RNA	Various unspecified Dicer1 mutations	Engineered cell line	Targeted therapy, Chemotherapy, Targeted therapy	Kinase inhibitor, Microtubule stabilizer, Kinase inhibitor	afatinib, docetaxel, bortezomib	TRUE	Resistance confers no significant intrinsic fitness difference	Resistant and parental lines have similar growth rates in monoculture.
Cooperative adaptation to therapy (CAT) confers resistance in heterogeneous non-small cell lung cancer (Craig et. al, 2019)	2	Resistance confers benefit	*in vitro*	3D co-culture spheroids	In co-culture, Dicer1 mutants have a growth advantage over sensitive clones in absence of drug	Population growth rate	Approximate clonal contribution by fluorescence imaging and absolute number of cells by flow cytometry	FALSE	Mouse non-small cell lung cancer	Lung and Chest	Dicer1 WT	Dicer1 M2 mutant	1:1	Noncoding RNA	Various unspecified Dicer1 mutations	Engineered cell line	Targeted therapy, Chemotherapy, Targeted therapy	Kinase inhibitor, Microtubule stabilizer, Kinase inhibitor	afatinib, docetaxel, bortezomib	TRUE	Resistance confers no significant intrinsic fitness difference	Resistant and parental lines have similar growth rates in monoculture.
Cooperative adaptation to therapy (CAT) confers resistance in heterogeneous non-small cell lung cancer (Craig et. al, 2019)	3	Resistance confers benefit	*in vitro*	3D co-culture spheroids	In co-culture, Dicer1 mutants have a growth advantage over sensitive clones in absence of drug	Population growth rate	Approximate clonal contribution by fluorescence imaging and absolute number of cells by flow cytometry	FALSE	Mouse non-small cell lung cancer	Lung and Chest	Dicer1 WT	Dicer1 M3 mutant	1:1	Noncoding RNA	Various unspecified Dicer1 mutations	Engineered cell line	Targeted therapy, Chemotherapy, Targeted therapy	Kinase inhibitor, Microtubule stabilizer, Kinase inhibitor	afatinib, docetaxel, bortezomib	TRUE	Resistance confers no significant intrinsic fitness difference	Resistant and parental lines have similar growth rates in monoculture.
Spatial heterogeneity and evolutionary dynamics modulate time to recurrence in continuous and adaptive cancer therapies (Gallaher et al., 2018)	1	Resistance is costly	*in vitro*	2D co-culture	In co-culture, doxorubicin-resistant MCF7 clones lose against sensitive MCF7 clones	Population growth rate	Absolute cell number by flow cytometry and fluorescence imaging	FALSE	Human ER+ breast cancer	Breast	MCF7	MCF7Dox	1:1	Efflux pumps	Pgp hi	Constant dosing In-vitro	Chemotherapy	Topoisomerase inhibitor	doxorubicin	TRUE	Resistance is intrinsically costly	Resistant lines had a much slower growth rate than sensitive lines in monoculture
A Strategy to Delay the Development of Cisplatin Resistance by Maintaining a Certain Amount of Cisplatin-Sensitive Cells (Duan et al., 2017)	1	Resistance is costly	*in vivo*	Orthotopic mouse model	1:1 HeLa:HeLa/ddp tumors displayed poor growth in vivo compared to sensitive-only tumors. No apparent growth rate change was observed for sensitive HeLa cells in mixed-culture tumors compared to sensitive-only tumors, but resistant cells were almost completely absent at 40 days post transplantation.	Population growth rate, Cellular proliferation rate, Cellular death rate	Tumor volume, cell cycle status by Ki67, and apoptosis rate by TUNEL staining	NA	Human cervical adenocarcinoma	Reproductive	HeLa-RFP	HeLa/ddp	1:1	Metabolic rewiring	Glutamine catabolism mediated by KRAS upregulation	Escalating dose In-vitro	Chemotherapy	Platinating agent	cisplatin	TRUE	Resistance is intrinsically costly	Sensitive-only tumors grew large, while resistant-only tumors remained at a small volume despite the same number of tumors cells being injected.
Spatial competition constrains resistance to targeted cancer therapy (Bacevic et al., 2019)	1	Resistance is costly	*in vitro*	2D co-culture	In co-culture, CDK inhibitor-resistant HCT116 cells experienced a growth disadvantage compared to sensitive clones	Population growth rate	Approximate clonal contribution by fluorescence imaging and absolute number of cells by flow cytometry	FALSE	Human colorectal cancer	Gastrointestinal	GFP+CDKi-sensitive HCT116	mCherry+R50 HCT116	1:1, 9:1, 99:1	Target modification	CDK2 mutation	Constant dosing In-vitro	Targeted therapy	Cell cycle inhibitor	NU6102	TRUE	Resistance is intrinsically costly	WT monocultures have a higher growth rate than res., suggesting intrinsic fitness penalty
Spatial competition constrains resistance to targeted cancer therapy (Bacevic et al., 2019)	2	Resistance is costly	*in vitro*	3D co-culture spheroids	In 3D co-culture, CDK inhibitor-resistant HCT116 cells experienced a growth disadvantage compared to sensitive clones	Population growth rate	Approximate clonal contribution by fluorescence imaging and absolute number of cells by flow cytometry	FALSE	Human colorectal cancer	Gastrointestinal	GFP+CDKi-sensitive HCT116	mCherry+R50 HCT116	99:1	Target modification	CDK2 mutation	Constant dosing In-vitro	Targeted therapy	Cell cycle inhibitor	NU6102	FALSE	NA	NA
Evolutionary approaches to prolong progression-free survival in breast cancer (Silva et al., 2012)	1	Resistance confers benefit	*in vitro*	2D co-culture	In high-glucose conditions, MCF7/Dox mutants had a slightly shorter doubling time than parental cells.	Population growth rate	Approximate cell number by crystal violet staining and fluorescence imaging	FALSE	Human ER+ breast cancer	Breast	MCF7	MCF7/Dox	2:1	Efflux pumps	Pgp hi	Escalating dose In-vitro	Chemotherapy	Topoisomerase inhibitor	doxorubicin	TRUE	Resistance confers no significant intrinsic fitness difference	Sensitive and resistant lines had similr growth rates in hi-glucose conditions.
Evolutionary approaches to prolong progression-free survival in breast cancer (Silva et al., 2012)	2	Resistance confers benefit	*in vitro*	2D co-culture	In low-glucose conditions, MCF7/Dox mutants had a slightly shorter doubling time than parental cells.	Population growth rate	Approximate cell number by crystal violet staining and fluorescence imaging	TRUE	Human ER+ breast cancer	Breast	MCF7	MCF7/Dox	2:1	Efflux pumps	Pgp hi	Escalating dose In-vitro	Chemotherapy	Topoisomerase inhibitor	doxorubicin	TRUE	Resistance is intrinsically costly	Sensitive lines had higher growth rates in low-glucose conditions compared to resistant lines, which had a sharp dropoff after 72h.
Adriamycin-resistant cells are significantly less fit than adriamycin-sensitive cells in cervical cancer. (Qi et al., 2021)	1	Resistance is costly	*in vivo*	Orthotopic mouse model	Mixed tumors consisting of 1:1 initial seeding ratio grew as large as fully parental tumors and consisted of nearly entirely parental cells by endpoint	Population growth rate	Tumor volume and approximate clonal contribution by fluorescence imaging	NA	Human cervical adenocarcinoma	Reproductive	HeLa-RFP	HeLa/ADR	1:1	NA	NA	Escalating dose In-vitro	Chemotherapy	Topoisomerase inhibitor	doxorubicin	TRUE	Resistance is intrinsically costly	Resistant-only tumors were far smaller than parental tumors
Dynamic Phenotypic Switching and Group Behavior Help Non-Small Cell Lung Cancer Cells Evade Chemotherapy. (Nam et al., 2022)	1	Resistance is costly	*in vitro*	2D co-culture	both 12-hour and 3-week co-cultures of H23 and H2009 cells resulted in a growth advantage of sens over res due to a secreted factor from sensitive cells	Population growth rate	Approximate clonal contribution by continuous fluorescence imaging	FALSE	Human non-small cell lung cancer	Lung and Chest	S (H23)	T (H2009)	8:1, 2:1, 1:1, 1:2, 1:8	NA	NA	Patient-derived	Chemotherapy	Platinating agent	cisplatin	FALSE	NA	NA

Title	Exp. #	Conclusion	Modality	Experimental model type	Summary	Measure of fitness	Fitness data collected by	Resource limited?	Cancer model	Cancer Type (ACS Class)	Sensitive line	Resistant line	Admixture ratio (Sens:Res)	Resistance mechanism category	Specific resistance characteristic	Resistance induction method	Treatment category	Class of drug	Trx name	Includes monoculture?	Monoculture Conclusion	Monoculture Summary
Paracrine behaviors arbitrate parasite-like interactions between tumor subclones (Noble et al., 2021)	1	Resistance is costly	*in vitro*	2D co-culture	"Two cell lines derived from a single mouse mammary carcinoma – 168 and 4T07 cells – have similar growth rates when cultured individually, yet the 4T07 clone displays a dominant phenotype when grown together"	Population growth rate, Cellular proliferation rate, Cellular death rate	Absolute number of cells and death rate by flow cytometry	FALSE	Mouse mammary carcinoma	Breast	4T07	168FARN	1:4, 1:3, 3:1, and 4:1	NA	NA	Escalating dose In-vitro	Chemotherapy	Nucleic acid synthesis Inhibitor	2,6-diaminopurine	TRUE	Resistance confers no significant intrinsic fitness difference	Similar growth rates in monoculture
E2F1 mediates competition, proliferation and response to cisplatin in cohabitating resistant and sensitive ovarian cancer cells (Valdivia et al., 2024)	1	Resistance is costly	*in vitro*	2D co-culture	Co-culture of OVCAR5 and resistant derivatives resulted in a growth advantage of sensitive cells at a penalty to res.	Population growth rate	Absolute number of cells by flow cytometry	FALSE	Human ovarian cancer	Reproductive	OVCAR5	OVCAR5 CisR	1:1, 1:2, 1:5, 1:7	NA	NA	Escalating dose In-vitro	Chemotherapy	Platinating agent	cisplatin	TRUE	Resistance is intrinsically costly	Sens cells grow faster in co-culture than in monoculture, and res cells grow slower
E2F1 mediates competition, proliferation and response to cisplatin in cohabitating resistant and sensitive ovarian cancer cells (Valdivia et al., 2024)	2	Resistance is costly	*in vitro*	2D co-culture	Co-culture of PE01 and resistant PE04 cells resulted in a growth advantage of sensitive cells at a penalty to res.	Population growth rate	Absolute number of cells by flow cytometry	FALSE	Human ovarian cancer	Reproductive	PE01	PE04	1:1, 1:2, 1:5, 1:7	NA	NA	Patient-derived	Chemotherapy	Platinating agent	cisplatin	TRUE	Resistance is intrinsically costly	Sens cells grow faster in co-culture than in monoculture, and res cells grow slower
E2F1 mediates competition, proliferation and response to cisplatin in cohabitating resistant and sensitive ovarian cancer cells (Valdivia et al., 2024)	3	Resistance is costly	*in vitro*	2D co-culture	Long-term co-culture of PE01 and resistant PE04 cells resulted in a growth advantage of sensitive cells at a penalty to res.	Population growth rate	Approximate clonal contribution by fluorescence imaging	FALSE	Human ovarian cancer	Reproductive	PE01	PE04	1:2	NA	NA	Patient-derived	Chemotherapy	Platinating agent	cisplatin	FALSE	NA	NA
Metronomic Chemotherapy Modulates Clonal Interactions to Prevent Drug Resistance in Non-Small Cell Lung Cancer. (Bondarenko et al., 2021)	1	Resistance is costly	*in vitro*	2D co-culture	Drug-sensitive A549 clones inhibit the proliferation of the drug-resistant A549/EpoB40 clones	Population growth rate	Approximate clonal contribution by fluorescence imaging	FALSE	Human non-small cell lung cancer	Lung and Chest	A549	A549/EpoB40	23:5	NA	NA	Escalating dose In-vitro	Chemotherapy, Chemotherapy	Platinating agent, Microtubule stabilizer	cisplatin, patupilone	TRUE	Resistance is intrinsically costly	Res cells grow slower in co-culture compared to monoculture
Metronomic Chemotherapy Modulates Clonal Interactions to Prevent Drug Resistance in Non-Small Cell Lung Cancer. (Bondarenko et al., 2021)	2	Resistance is costly	*in vitro*	2D co-culture	Drug-sensitive HT29 clones inhibit the proliferation of the drug-resistant HT29/Rox1 clones	Population growth rate	Approximate clonal contribution by fluorescence imaging	FALSE	Human colorectal cancer	Gastrointestinal	HT29	HT29/Rox1	23:5	NA	NA	Escalating dose In-vitro	Chemotherapy	Platinating agent	oxaliplatin	FALSE	NA	NA
Metronomic Chemotherapy Modulates Clonal Interactions to Prevent Drug Resistance in Non-Small Cell Lung Cancer. (Bondarenko et al., 2021)	3	Resistance is costly	*in vitro*	2D co-culture	Drug-sensitive A549 clones inhibit the proliferation of the drug-resistant A549/EVP16 clones	Population growth rate	Approximate clonal contribution by fluorescence imaging	FALSE	Human non-small cell lung cancer	Lung and Chest	A549	A549/VP16	23:5	NA	NA	Escalating dose In-vitro	Chemotherapy	Topoisomerase inhibitor	etoposide	FALSE	NA	NA
Metronomic Chemotherapy Modulates Clonal Interactions to Prevent Drug Resistance in Non-Small Cell Lung Cancer. (Bondarenko et al., 2021)	4	Resistance does not confer a significant fitness difference	*in vivo*	Orthotopic mouse model	"tumor cell composition did not change over time with a mixture of drug-sensitive A549-mtDsRed and drug-resistant A549/EpoB40-GFP"	Population growth rate	Absolute number of cells by flow cytometry	NA	Human non-small cell lung cancer	Lung and Chest	A549	A549/EpoB40	7:3	NA	NA	Escalating dose In-vitro	Chemotherapy, Chemotherapy	Platinating agent, Microtubule stabilizer	cisplatin, patupilone	FALSE	NA	NA
HSP90a promotes the resistance to oxaliplatin in HCC through regulating IDH1-induced cell competition (Wang et al., 2024)	1	Resistance confers benefit	*in vitro*	2D co-culture	Oxaliplatin resistance mediated by lipid metabolism allows resistant cells to win in co-culture	Population growth rate	Absolute cell number by flow cytometry and fluorescence imaging	FALSE	Human hepatocellular carcinoma	Gastrointestinal	MHCC97H-GFP	MHCC97H-OXR	1:1	Metabolic rewiring	Increased lipid metabolism	Escalating dose In-vitro	Chemotherapy	Platinating agent	oxaliplatin	FALSE	NA	NA
Establishment and Molecular Characterization of an In Vitro Model for PARPi-Resistant Ovarian Cancer (Klotz et al., 2023)	1	Resistance is costly	*in vitro*	2D co-culture	"In the co-culture setting, we observed that Olres-UWB cells had a clear competitive disadvantage compared to PARPi-sensitive UWB cells"	Population growth rate	Absolute number of cells by flow cytometry	FALSE	Human ovarian cancer	Reproductive	tdTomato-UWB1.289	eGFP-Olres-UWB1.289	1:1	EMT	EMT phenotype	Escalating dose In-vitro	Targeted therapy	PARP Inhibitor	olaparib	TRUE	Resistance confers no significant intrinsic fitness difference	"There was no significant difference in basal proliferation between PARPi-resistant Olres-UWB vs. parental PARPi-sensitive cells in monocultures"
Establishment and Molecular Characterization of an In Vitro Model for PARPi-Resistant Ovarian Cancer (Klotz et al., 2023)	2	Resistance is costly	*in vitro*	2D co-culture	"We observed similar clonal dynamics in this model, including a competitive disadvantage of Olres-UWB+BRCA1 under drug-free conditions"	Population growth rate	Absolute number of cells by flow cytometry	FALSE	Human ovarian cancer	Reproductive	tdTomato-UWB1.289+BRCA1	eGFP-Olres-UWB1.289+BRCA1	1:1	EMT	EMT phenotype	Escalating dose In-vitro	Targeted therapy	PARP Inhibitor	olaparib	TRUE	Resistance is intrinsically costly	Resistant lines slightly lower growth rate in monoculture
Evolutionary dynamics of cancer multidrug resistance in response to olaparib and photodynamic therapy (Baglo et al., 2021)	1	Resistance is costly	*in vitro*	2D co-culture	"The sensitive variant OVCAR-8-DsRed2 quickly begins dominating the population within days of culture and is at around 95%"	Population growth rate	Approximate clonal contribution by fluorescence imaging and absolute number of cells by flow cytometry	FALSE	Human ovarian cancer	Reproductive	OVCAR-8-DsRed2	NCI/ADR-RES-EGFP	1:1	Efflux pumps	Pgp hi	Escalating dose In-vitro	Targeted therapy	PARP Inhibitor	olaparib	TRUE	Resistance is intrinsically costly	Resistant lines slightly lower growth rate in monoculture
